# ART and AutoART ECAP measurements and cochlear nerve anatomy as predictors in adult cochlear implant recipients

**DOI:** 10.1007/s00405-023-08444-5

**Published:** 2024-01-14

**Authors:** Leonhard Schrank, Pascal Nachtigäller, Joachim Müller, John-Martin Hempel, Martin Canis, Jennifer L. Spiegel, Tobias Rader

**Affiliations:** 1 Division of Audiology, Department of Otorhinolaryngology, LMU University Hospital, LMU Munich, Marchioninistr. 15, 81377 Munich, Germany; 2 Department of Otorhinolaryngology, LMU University Hospital, LMU Munich, Munich, Germany; 3 German Center for Vertigo and Balance Disorders, LMU University Hospital, LMU Munich, Munich, Germany

**Keywords:** Cochlear implant, Cochlear nerve, Electrically evoked compound action potentials, Magnetic resonance imaging, Prognostic factors

## Abstract

**Purpose:**

The purpose of this retrospective study is to compare the results of electrically evoked compound action potential (ECAP) measurements using automatic auditory response telemetry (AutoART) with those obtained by ART in adults. The study also aimed to evaluate the predictive value of intraoperative ART and AutoART ECAPs for speech intelligibility (SI) and hearing success (HS), and to determine if cochlear nerve (CN) cross-sectional area (CSA) obtained preoperatively by magnetic resonance imaging (MRI) scans could predict ART and AutoART ECAPs and SI and HS outcome.

**Methods:**

The study analyzed and correlated ART and AutoART ECAP thresholds at electrodes E2, E6, and E10, as well as averaged ECAP thresholds over electrodes E1–E12, using data from 32 implants. Correlations were also examined for ART and AutoART ECAP slopes. In addition, averaged ART and AutoART ECAP thresholds and slopes over all 12 electrodes for each participant were correlated with CN CSA measured from MRI sequences. SI of the monosyllabic Freiburg Speech Test at 65 dB sound pressure level was examined along with averaged ART and AutoART thresholds and slopes over all 12 electrodes. A parallel analysis was performed for HS, derived from the difference between baseline and 6-month SI. Finally, correlations between CN CSA and SI, as well as CN CSA and HS were examined.

**Results:**

The results of the study showed a significant positive correlation between ART and AutoART ECAP thresholds and as well as slopes for E2, E6, E10 and averaged thresholds and slopes of E1–E12. However, no significant correlation was observed between ART and AutoART averaged ECAP thresholds and slopes and either SI and HS or CN CSA. Furthermore, no significant correlation was found between CN CSA and SI and HS.

**Conclusion:**

While AutoART is a reliable and safe program for measuring ECAPs in adults, the study found no preoperative prognostic information on intraoperative ECAP results using parameters extracted from current MRI sequences or pre-/intraoperative information on subsequent hearing outcome using ECAP and CN CSA.

## Introduction

A cochlear implant is a viable option for treating patients with severe to profound sensorineural hearing loss as it can partially restore their hearing. The CI system consists of a retroauricular audio processor that detects sounds and speech, and transmits them as electrical impulses via a coil to the implant anchored in the temporal bone. The implant, equipped with intracochlear electrodes, directly stimulates type 1 spiral ganglion cells (SGCs), which are the bipolar myelinated neurons of the cochlear nerve (CN) with afferent contacts to the inner hair cells. The location of these electrodes and the electrophysiological function of the CN are objectively assessed using electrically evoked compound action potentials (ECAPs) [2–4].

ECAPs are recorded using auditory nerve telemetry, marketed by MED-EL GmbH (Innsbruck, Austria) as auditory response telemetry (ART) [5]. An improved and enhanced version called AutoART is now available [6], which offers several advantages over ART, including autonomous measurement and automatic interruption during ECAP detection, resulting in easier handling and shorter measurement procedures [7]. Although AutoART is increasingly used in clinical practice, there are only a few studies comparing the ECAPs of ART and AutoART in different patient populations. For example, while a recent study compared these methods in infants and young children [8], no similar study has been performed in adults.

Several previous studies have investigated the relationship between ECAP measures and speech intelligibility (SI) in CI users. While some studies have found a strong correlation between the two [3, 9–11], others have failed to replicate these findings [12–14]. Overall, ECAP measures have been found to be unpredictable and inconsistent as a prognostic factor for SI [15]. However, these studies were all conducted using auditory nerve telemetry from Cochlear Corporation (Sydney, Australia), known as Nerve Response Telemetry (NRT) [16] and/or AutoNRT [17, 18]. AutoNRT requires the use of multiple ECAP responses at a given stimulus level to suppress noise, while using relatively large current steps to limit the duration of the measurement, which in turn reduces the accuracy of the ECAP measurement [19]. With AutoART, ECAP measurements are sampled with smaller stimulus steps and are therefore more accurate [19]. Another difference is the methodology for removing the stimulus artifact. ART uses the alternating polarity paradigm, while NRT allows the user to choose between alternating polarity and the subtraction method with a two-pulse forward-masking paradigm [20]. As alternating polarity introduces artifacts resulting from differing ECAP responses to anodic-leading and cathodic-leading pulses, additional deviations are introduced here. A more detailed discussion of differences in artifact rejection methods is presented in the paper of He et al. [4]. However, no study to date has compared SI with ART and AutoART ECAP measurements.

Another important prognostic factor for postoperative speech perception after cochlear implantation may be the CN cross-sectional area (CSA). The influence of its bipolar neurons, the SGCs, on speech perception has been controversially discussed in many studies [21–24]. It should be noted, however, that previous studies have only histologically examined, counted and correlated SGCs with SI in selected temporal bones. Physiologically, the SI encompasses the entire cochlea, where incoming acoustic signals are bundled by the SGCs of the different cochlear sections and transmitted to the CN. Since the SGCs can only be examined postmortem, it is necessary to find another way to make predictions preoperatively. As the CN is typically evaluated using magnetic resonance imaging (MRI) before surgery, it would be intriguing to investigate its potential on the SI. However, to the best of the authors’ knowledge this topic has not yet been explored.

Additionally, preoperative MRI sequences may offer insights into the relationship between CN and ECAP measurements, but this aspect remains unexplored in previous research.

Considering these findings and considerations, this retrospective study aims to accomplish three primary objectives in adults. First, to analyze ART and AutoART ECAPs and determine correlations between ART and AutoART ECAP thresholds and between ART and AutoART ECAP slopes in MED-EL subjects. Second, to correlate ECAP thresholds separately for ART and AutoART and ECAP slopes separately for ART and AutoART with CN CSA, SI, and Hearing Success (HS). Third, to examine the correlation between CN CSA and SI, and CN CSA and HS.

## Materials and methods

### Patient selection

This retrospective, monocentric study initially included 51 participants with severe to profound hearing loss who underwent unilateral or bilateral cochlear implantation at our tertiary academic center between 01/2020 and 05/2021.

Data were collected from digital patient clinical records, including surgical reports, medical history, treatment history, MRI scans, audiometric testing, and cochlear implant fitting software.

All patients over 18 years of age with severe to profound hearing loss who received CIs were included. Participants who did not have successful ECAP responses as measured by ART and/or AutoART, those with missing or inadequately resolved MRI sequences for CN CSA measurement, and those with language difficulties preventing completion of the German language test were excluded from the study.

### Electrically evoked compound action potential (ECAP) measurements (electrophysiological measurements)

Using a MAX Coil S and a MAX Interface Box (V1.0.0/V1.1.2), the CI was connected intraoperatively to the MAESTRO software (MED-EL, Innsbruck, Austria, version 9.0.4) for intraoperative ECAP recordings with both ART and AutoART.

In this study, the number of successfully registered intraoperative ECAP responses at each electrode (E1–E12) for both ART and AutoART were retrospectively evaluated and compared for each implant. An ECAP was considered successful if an ECAP response was registered at all 12 electrodes. In addition, the existing ECAP responses of each electrode were also examined and evaluated for their biphasic morphology, consisting of a negative (N1, 0.3–0.4 ms post stimulus) and a positive peak (P2, 0.6–0.7 ms post stimulus) [25]. If more than half of all 12 electrodes and more than half of the different stimulation intensities of each electrode showed a distinct biphasic waveform, this was classified as a “good ECAP response”. If predominantly truncated negative peaks due to an ECAP response below 0.3 ms and/or flattened positive peaks were observed using the above observation criteria, this was classified as a “questionable ECAP response”. A “poor ECAP response” was classified primarily by the observation criteria of missing or absent negative and positive peaks, but at least one electrode showed an ECAP response. In cases where the ART P2 values were considered ambiguous by the software, a manual adjustment in the range of 0.6–0.7 ms was used. However, this manual adjustment was not possible in the AutoART system because the software did not support manual adjustments.

For a more detailed analysis of ART and AutoART, the ECAP thresholds and slopes of the two measurement paradigms were compared and evaluated.

The software automatically generates amplitude growth functions (AGF) with a sigmoid curve composed of the interpeak differences (amplitude) between N1 and P2 at different stimulation intensities. The ECAP threshold is then defined as the intersection of the ECAP slope with the baseline (zero line), while the ECAP slope marks the steepest part of the sigmoid curve within the two inflection points where the curve flattens due to saturation of the neurons before and after the point.

For the analysis and correlation of ECAP thresholds and slopes for ART and AutoART, electrode 2 (E2) was selected for the apical electrodes, electrode 6 (E6) for the medial electrodes, and electrode 10 (E10) for the basal electrodes of the cochlea. Additionally, all averaged electrodes for E1 through E12 were evaluated for ART and AutoART ECAP thresholds and slopes.

For the analysis involving CN CSA and SI results, the ECAP thresholds and slopes for each subject were averaged over all 12 electrodes, as auditory speech perception and CN stimulation are typically distributed across all 12 electrodes.

### MRI-sequencing and measurement of the cochlear nerve

Routine preoperative MRI scans of the temporal bone were obtained retrospectively from the picture archiving and communication system (PACS) to measure CN CSA. As these scans were acquired outside of our hospital, they were obtained using different MRI scanners with varying technical specifications. Table 1 lists the scanners used for each subject, along with their respective field strengths, acquisition protocols, and image resolutions.

**Table 1:** Technical and physical parameters of MRI sequences used in the study

Scanner Type	TE (ms)	TR (ms)	ST (mm)	GAP (mm)	FOV (mm)	Matrix	ETL	RBW (kHz)	FA (°)
Siemens 1.0 T	5.6	11.2	1.0		75	320/240	1	130	70
Siemens 1.5 T	2.4–269.0	5.8–1300.0	0.4–0.8		75–100	256–512/287–384	1	130–455	62–150
Siemens 3.0 T	2.5–145.0	5.8–1000.0	0.4–0.7		91–00	320–448/307–448	1–71	285–422	47–110
Philips 1.5 T	110.0–297.8	1500.0–5563.0	1.1–5.0	0.6–5.5	80–125	219–368/166–278	14–171	115–1000	90
Philips 3.0 T	200	1500	1	1	100	376/270	40	163	90

Table provides an overview of the technical and physical parameters of the MRI sequences used in the study. The table includes information such as the scanner type, echo time (TE), pulse repetition time (TR), slice thickness (ST), interslice space (GAP), field of view (FOV), matrix size, echo train length (ETL), receiver bandwidth (RBW), and flip angle (FA). The range or specific values for each parameter are listed accordingly. This table presents the key technical details of the MRI sequences utilized in the study

Three-dimensional constructive interference in the steady state (3D CISS) sequences from Siemens scanners (Siemens AG, Munich, Germany), and balanced Fast Field Echo (bFFE) sequences from Philips scanners (Philips N.V., Amsterdam, The Netherlands) were used in this study at different magnetic field strengths (T 1.0, T 1.5, T 3.0). These sequences are known for their high-resolution imaging of fine structures such as cranial nerves and inner ear.

**Fig. 1:**
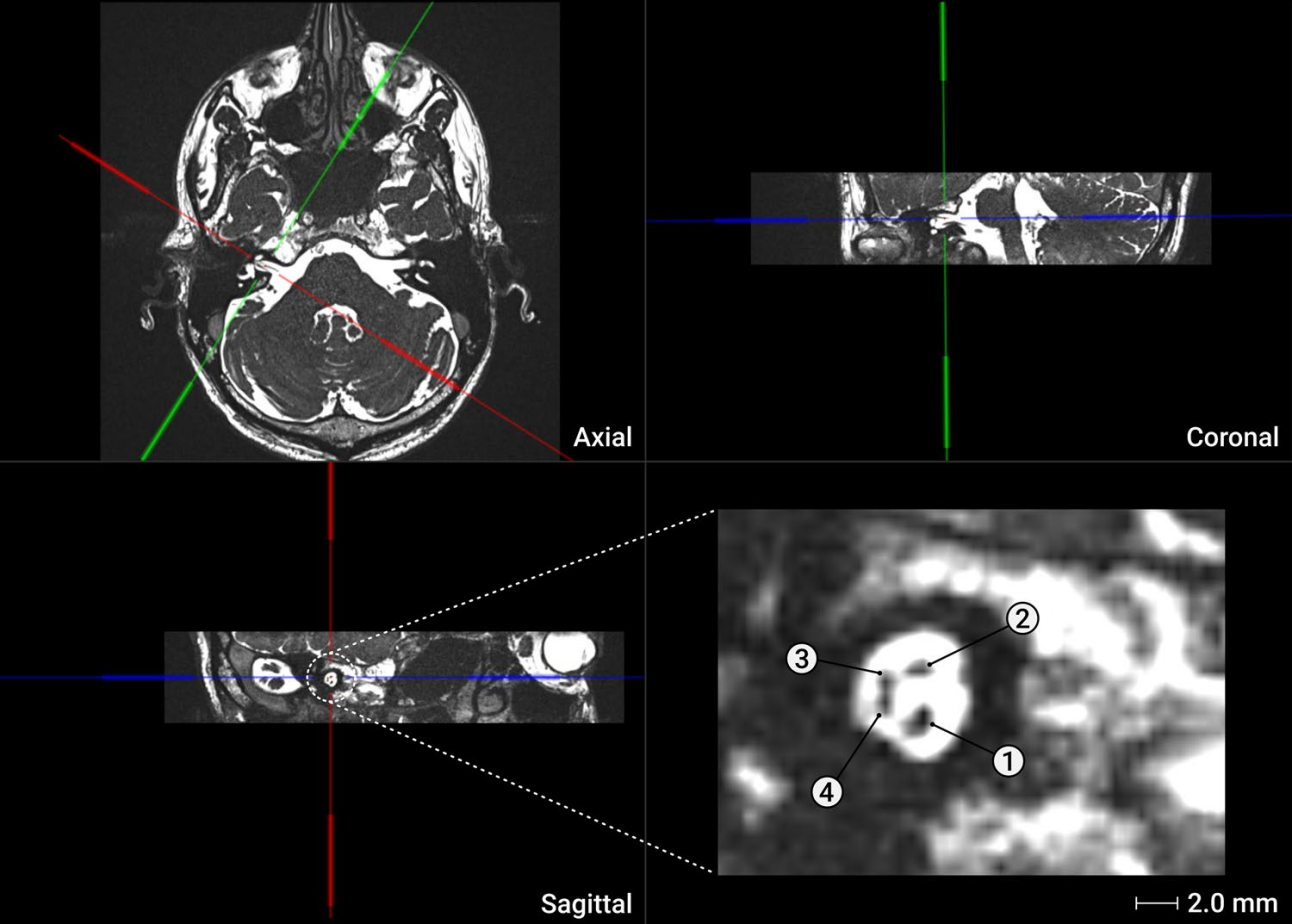
Cochlear and facial nerve measurement. A 3D CISS MRI sequence was employed to examine the petrous bone of a 45-year-old adult. The resulting image was aligned axially, coronally, and sagittally, revealing an enlarged right internal auditory canal housing the cochlear nerve (1), facial nerve (2), and superior (3) and inferior vestibular nerves (4). Software tools were utilized to measure the longest and widest segments of the cochlear and facial nerves. This figure description is adapted from OTOPLAN software (CAScination AG, Bern, Switzerland, version 3)

3D CISS and bFFE sequences were imported as DICOM (Digital Imaging and Communications in Medicine) data into the tablet-based software OTOPLAN (CAScination AG, Bern, Switzerland, version 3) (CE certification number: G1 17 10 95657 003). A detailed description of the software can be found in Spiegel et al. [26]. The software was used to align and arrange the axial, coronal, and sagittal slices of the implanted ear to visualize the hyperintense internal auditory canal (IAC) and its hypointense neural pathways. The IAC was positioned and aligned in the sagittal plane of section until the facial nerve (FN), the cochlear nerve (CN), the superior vestibular nerve (SVN), and the inferior vestibular nerve (IVN) were ideally clearly delineated. However, in most cases, only the CN and FN were clearly visible, while the two VNs were often fused into a single crescent-shaped unit (Fig. 1).

Like our previous study in children [8], a software measurement tool was used to determine the longest and widest section of the CN and FN in three passes to reduce measurement error. The CSA in square millimeters was then calculated from the mean values using the formula for elliptical areas (*π* × ½ length × ½ width).

The CN CSA was compared to the FN CSA in terms of degeneration due to long-term SNHL. In previous studies, CN was found to be greater than or equal to the FN in 90% of cases and was therefore considered normative [27, 28]. Hypoplasia was defined as a CN CSA smaller than the FN CSA, while aplasia was defined as the absence of the CN in the MRI sequences.

### Audiometric data

In this study, we retrospectively analyzed the performance on the Freiburg speech test, standardized according to DIN 45621-1 and DIN 45626-1 [29], consisting of 20 German monosyllabic nouns (“Freiburger Einsilbertest”), at 65 dB SPL (sound pressure level). A CCITT (Commité Consultatif International Télégrafique et Téléfonique) noise masker was used to occlude the unaided ear when necessary to prevent overhearing in that ear. We assessed both absolute speech intelligibility (SI) and hearing success (HS) for monosyllables at 65 dB SPL at 6 months. HS was defined as the difference in absolute hearing scores for monosyllabic words between 6 months and baseline at the initial fitting.

### Data analysis

Statistical analysis of the data was performed using Microsoft Excel (Microsoft, Redmond, WA, USA, version 2110) and Statistical Package for Social Sciences (SPSS) software (IBM, Armonk, NY, USA, version 28). Descriptive statistics were used to present the results of this study.

The ART and AutoART ECAP thresholds and slopes E2/E6/E10 and averaged ECAP thresholds and slopes E1–E12 were analyzed and correlated. In addition, the ART and AutoART ECAP thresholds and slopes averaged over all 12 electrodes for each subject were correlated with the CN CSA and SI and HS. The CN CSA was also correlated with SI and HS.

The normality of the data was tested using the Shapiro–Wilk normality test. The paired samples *t*-test was used to analyze the means of ART and AutoART ECAP thresholds and slopes, except for ART and AutoART ECAP slopes E6/E10, which had non-normal distribution, and therefore the Wilcoxon signed-rank test was used to examine central tendency.

Pearson's correlation coefficient was used for interval-scaled and linear variables, whereas Spearman's rank correlation coefficient was used for the correlation between CN CSA and ART and AutoART ECAP thresholds and slopes averaged for each subject and between CN CSA and SI and HS due to the nonlinearity of the measures.

Statistical significance was set at *p* < 0.05.

## Results

### Patient selection

19 male and 13 female adult participants were recruited for this retrospective study, after applying exclusion criteria. One individual received bilateral implantation during the observation period. The study included 33 ears/implants, 19 on the right and 14 on the left.

The mean age of these participants at the time of implantation was 54.63 years (20–89 years). 20 participants were implanted with MED-EL Synchrony 2, and 13 were implanted with Synchrony 2 S-Vector cochlear implants. The electrode arrays used were "Standard" in 10 participants, "FlexSoft" in 12, and "Flex28" in 11.

The duration of deafness was less than 2 years in 2 subjects, 2–5 years in 6 subjects, 10 years in 1 subject, and more than 10 years in 3 subjects. The duration of deafness was unknown in the remaining 20 subjects.

The etiologic causes of deafness in the subjects were acoustic trauma (*n* = 3), genetic/hereditary (*n* = 3), Meniere's disease (*n* = 3), infections such as meningitis (*n* = 3), prematurity (*n* = 2), and hearing loss (*n* = 1). The cause of deafness was unknown in the remaining 17 subjects.

### Electrically evoked compound action potential (ECAP) measurements

**Fig. 2:**
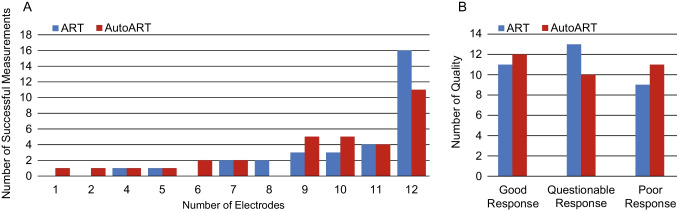
Success and quality of electrically evoked compound action potentials. **A** Presents a comparison between the successful measurements of electrically evoked compound action potentials (ECAPs) obtained by two different methods: auditory response telemetry (ART) and AutoART. Successful measurements are defined as the maximum number of measurements obtained across all electrodes, as shown in the graph on the right. The left side shows the number of electrodes with fewer successful measurements. The results are visually represented using blue for ART and red for AutoART. **B** Illustrates the comparison between the response quality of ART (blue depicted) and AutoART (red depicted) ECAPs. Responses were categorized as good, questionable, or poor based on the presence of a typical biphasic ECAP morphology

The number of electrodes with successful ECAP responses varied from 12 electrodes, representing the entire array and thus the highest success rate, to a single electrode (Fig. 2A). Notably, while 16 implants achieved a full array in ART, only 11 implants reached this level in AutoART. For 11 electrodes, successful ECAP responses were observed in 4 implants for both measurement paradigms. In addition, ART showed successful ECAP responses in 10 and 9 electrodes in 3 implants each, in 8 and 7 electrodes in 2 implants each, and in 5 and 4 electrodes in 1 implant each. AutoART, on the other hand, showed successful ECAP responses at 10 and 9 electrodes for 5 implants each, 7 and 6 electrodes for 2 implants each, and 5, 4, 2, and 1 electrode for 1 implant each.

The specific electrodes that showed superior performance in ART and AutoART in terms of individual electrode functionality remain uncertain. For ART, the average number of successful ECAP responses per electrode varied by implant from 24 (for E1), 25 (for E2), 27 (for E3/E4), 30 (for E5), 28 (for E6), 29 (for E7/E8), 31 (for E11), and 29 (for E12). For AutoART, the average number of successful ECAP responses per electrode was 22 (for E1), 23 (for E2), 25 (for E3-E6), 24 (for E7), 25 (E8), 28 (E9), 27 (E10), 25 (E11), and 26 (E12) for all implants.

As shown in Fig. 2B, AutoART (12) showed slightly better performance than ART (11) in producing good ECAP responses, but also resulted in proportionally poorer ECAP quality in 11 implants compared to ART (9). In contrast, ART produced more questionable responses than AutoART (10) in 13 implants.

Mean ECAP thresholds for ART and AutoART were compared for electrodes E2, E6, and E10. The mean ECAP threshold for ART was 12.00 ± 3.61 qu (5.28–21.36 qu; 1 qu ≈ 1 nC) for E2, 10.27 ± 3.91 qu (4.09–20.89 qu) for E6, and 11.98 ± 4.64 qu (3.14–21.01 qu) for E10. In contrast, the mean ECAP threshold for AutoART was 14.55 ± 3.41 qu (10.08–21.25 qu) for E2, 14.76 ± 3.01 qu (8.81–19.55 qu) for E6, and 17.00 ± 4.14 qu (8.56–23.55 qu) for E10.

A paired sample *t* test was used to test for significant differences in ECAP thresholds and slopes between the ART and AutoART measurement paradigms. The results showed that the mean ART ECAP thresholds for E2 were significantly lower than the AutoART ECAP thresholds for the same electrode (*t*(21) = − 4.79; *p* = 0.001) with a strong effect size *d* = 1.02 according to Cohen's guidelines [30]. Comparable results were found for electrodes E6 (*t*(22) = − 7.13; *p* = 0.001; *d* = 1.49) and E10 (*t*(26) = − 8.04; *p* = 0.001; *d* = 1.55). Furthermore, the *t* test revealed a significant difference between the averaged ART and AutoART thresholds of all 12 electrodes (*t*(11) = − 12.04; *p* = 0.001, *d* = 3.48).

The mean ART ECAP slope was 44.23 ± 25.71 µV/qu (12.32–103.80 µV/qu) for E2, 23.09 ± 10.62 µV/qu (10.29–51.60 µV/qu) for E6, and 22.37 ± 12.83 µV/qu (5.85–49.33 µV/qu) for E10. For AutoART, the mean ECAP slope was 45.08 ± 27.33 µV/qu (7.72–105.39 µV/qu) for E2, 24.94 ± 11.15 µV/qu (7.32–55.65 µV/qu) for E6, and 22.71 ± 12.67 µV/qu (6.02–54.58 µV/qu) for E10.

For ECAP slopes, *t*-test results showed no significant difference in means between ART and AutoART for E2 (*t*(21) = − 0.48; *p* > 0.05; *d* = 0.10). Similarly, the Wilcoxon signed-rank test showed no significant difference in the medians of the ECAP slopes for E6 between ART and AutoART (*z* = − 1.28; *p* > 0.05; *n* = 23), with a small Cohen's median effect size (*r* = 0.27). For E10, no significant difference was found between ART and AutoART (*z* = − 0.37; *p* > 0.05; *n* = 27; *r* = 0.07). However, when the ECAP slopes of electrodes E1–E12 were averaged, the *t*-test showed a significant difference in means between ART and AutoART (*t*(11) = − 2.53; p = 0.03; d = 0.73), indicating that the averaged ECAP slopes of ART were significantly lower than those of AutoART.

**Fig. 3:**
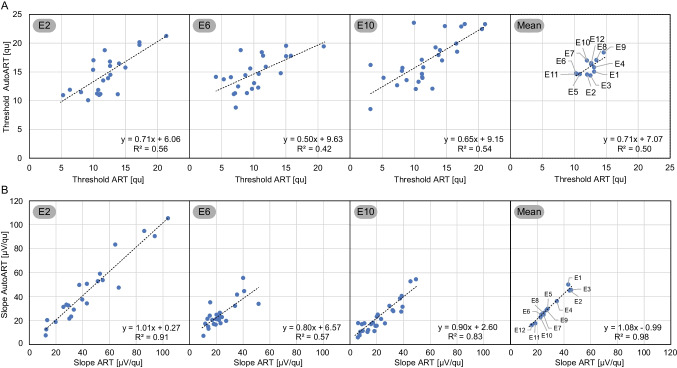
Correlation of electrically evoked compound action potential thresholds and slopes between auditory response telemetry (ART) and AutoART. The first three graphs above show the correlation of electrically evoked compound action potential (ECAP) thresholds between ART (*x*-axis) and AutoART (*y*-axis) for individually selected electrodes: E2 for the basal, E6 for the medial and E10 for the apical regions of the cochlea. The last top graph represents the average of all 12 electrodes examined. The lower plots show the same correlation between ART and AutoART for the above electrodes, but with ECAP slopes

The correlation between ART and AutoART for ECAP thresholds and slopes was analyzed using Pearson's correlation coefficient. Figure 3A shows a strong positive correlation between ART and AutoART ECAP thresholds for E2 (*r* = 0.75; *p* = 0.001), E6 (*r* = 0.65; *p* = 0.001), and E10 (*r* = 0.74; *p* = 0.001). A similarly strong positive correlation was observed for the averaged ECAP thresholds of all 12 electrodes (*r* = 0.70; *p* = 0.011). Figure 3A also shows that the ECAP thresholds for E1 to E5 were below the regression line, while those for E6 to E12 were above the line.

The correlation analysis between ART and AutoART for ECAP slopes yielded even stronger results compared to the ECAP thresholds of the two measurement paradigms. The correlations were high and significant for E2 (*r* = 0.95; *p* = 0.001), E6 (*r* = 0.76; *p* = 0.001), and E10 (*r* = 0.91; *p* = 0.001), while the strongest positive correlation was observed for the averaged slope of all 12 electrodes (*r* = 0.99; *p* = 0.001). Figure 3B shows that the ECAP slope data had relatively little scatter and closely followed the correlation line. Interestingly, the ECAP slopes decreased progressively from the apical to the basal electrodes, resulting in a flattened slope curve. This trend is most evident in the averaged ECAP slopes of all 12 electrodes, with the apical electrodes at the top of the correlation line, the medial electrodes in the middle, and the basal electrodes at the bottom.

### Correlation of ECAP responses with speech intelligibility

To evaluate the relationship between ECAP responses and speech perception, we first correlated the averaged ECAP thresholds and slopes for ART and AutoART across all 12 electrodes for each subject with the absolute speech intelligibility (SI) at the 6-month fitting and second with the hearing score (HS) for monosyllables presented at 65 dB SPL. On average, 31 subjects had an absolute SI of 40.2 ± 25.4% (0–85%) at the 6-month fitting, while 19 subjects had a HS of 30.0 ± 27.6% (− 35 to 85%).

**Fig. 4:**
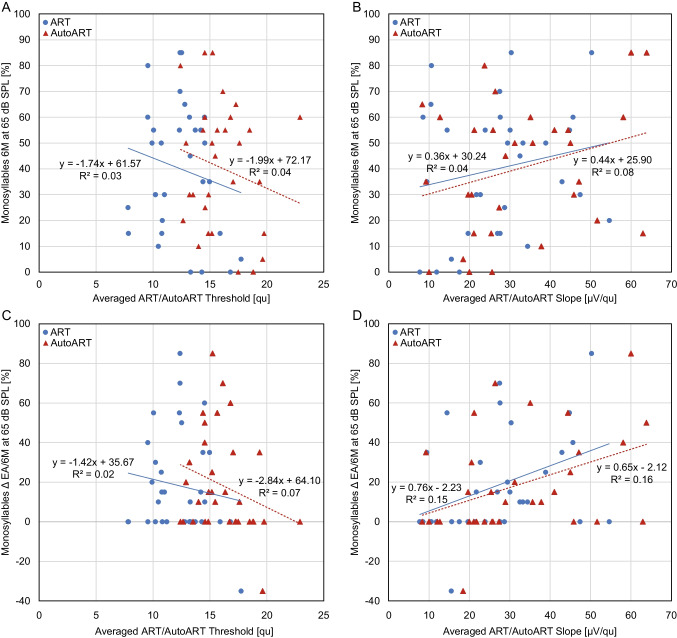
Correlation of electrically evoked compound action potential thresholds/slopes and speech intelligibility. The upper figures depict the correlations between electrically evoked compound action potential (ECAP) for auditory response telemetry (ART)/AutoART averaged over all 12 electrodes for each subject and monosyllabic speech intelligibility at a sound pressure level (SPL) of 65 dB (*y*-axis) at the 6-month mark after initial cochlear implant (CI) fitting. **A** Presents the comparison of ART (red depicted)/AutoART (blue depicted) ECAP thresholds (*x*-axis), while **B** represents this comparison for ART/AutoART ECAP slopes (*x*-axis). The lower figures display the correlation of ART (red)/AutoART (blue) ECAP thresholds (**C**) and slopes (**D**) (both *x*-axis) with the monosyllabic hearing success at 65 dB SPL (*y*-axis), as measured by the difference between the initial fitting and the 6-month follow-up evaluation

Spearman's rank correlation coefficient was used to examine the relationship between HS for monosyllables at 65 dB SPL and averaged ECAP thresholds and slopes for ART and AutoART across all 12 electrodes. No significant correlations were found between the HS and ECAP thresholds of ART (*ρ* = − 0.3; *p* > 0.05) and AutoART (*ρ* = − 0.3; *p* > 0.05) (Fig. 4B). Similarly, no significant correlations were found between HS and the ECAP slopes of ART (*ρ* = 0.1; *p* > 0.05) and AutoART (*ρ* = − 0.1; *p* > 0.05) (Fig. 4C). These results indicate that there is no significant relationship between HS for monosyllables at 65 dB SPL and ECAP responses.

For HS, Spearman's rank correlation coefficient showed no significant correlation between the averaged ECAP thresholds of ART (*ρ* = − 0.2; *p* > 0.05) and AutoART (*ρ* = − 0.2; *p* > 0.05). There was no significant correlation between the averaged ECAP slopes of ART (*ρ* = 0.3; *p* > 0.05) and AutoART (*ρ* = 0.4; *p* > 0.05) with HS (Fig. 4B).

### Sequencing and measurement of the cochlear nerve

CN CSA was compared with FN CSA in 33 implanted ears. The mean CN CSA was 1.59 ± 0.32 mm^2^ (1.00–2.22 mm^2^), while the mean FN CSA was 1.04 ± 0.18 mm^2^ (0.73–1.40 mm^2^). CN CSA was larger than FN CSA in 97% (*n* = 32) of the ears, indicating a normal size relationship. In only 3% (*n* = 1) of the ears, the CN CSA was smaller than the FN CSA, indicating a hypoplastic CN. No aplastic CN was found in any of the 33 ears.

### Correlation of ECAP responses with the cochlear nerve

**Fig. 5:**
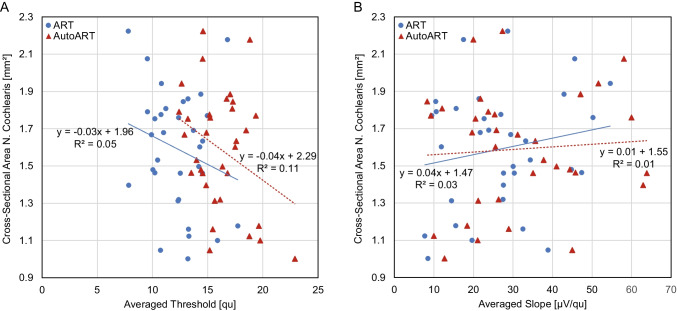
Correlation of electrically evoked compound action potential and cochlear nerve cross-sectional area. **A** Shows the correlations between the auditory response telemetry (ART) (blue depicted) and AutoART (red depicted) electrically evoked compound action potential (ECAP) thresholds (*x*-axis), averaged over all 12 electrodes for each participant, and the preoperatively measured cochlear nerve (CN) cross-sectional area (CSA) (*y*-axis). **B** Shows the correlation between ART/AutoART ECAP slopes (*x*-axis), averaged over all 12 electrodes for each participant, and CN CSA (*y*-axis)

Figure 5A shows the results of Spearman's rank correlation coefficient examining the relationship between ECAP values averaged over all 12 electrodes and CN CSA. For both ART and AutoART, the correlation coefficients were not significant (ART: *ρ* = − 0.18, *p* > 0.05; AutoART: *ρ* = − 0.23, *p* > 0.05). As seen in Fig. 5B, the correlation of CN CSA with ECAP slopes was not significant for ART (*ρ* = 0.12, *p* > 0.05) and for AutoART (*ρ* = 0.01, *p* > 0.05).

### Correlation of the cochlear nerve with speech intelligibility

**Fig. 6:**
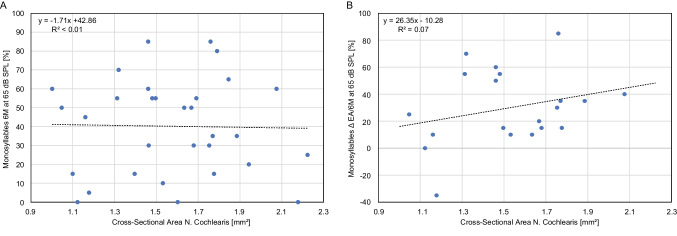
Correlation of cochlear nerve cross-sectional area and speech intelligibility. **A** Depicts the correlations between preoperatively measured cross-sectional area (CSA) of the cochlear nerve (CN) (*x*-axis) and monosyllabic speech intelligibility at a sound pressure level (SPL) of 65 dB (*y*-axis) during the 6-month follow-up period after the initial fitting of a cochlear implant (CI). **B** Illustrates the correlation between CN CSA (*x*-axis) and the level of hearing success for monosyllabic words at 65 dB SPL (*y*-axis), as quantified by the difference between the initial fitting and the 6-month follow-up evaluation

Spearman's rank correlation coefficient was used to evaluate the correlation between CN CSA and SI measured after 6 months of fitting for monosyllabic words (Fig. 6A). The result showed no correlation (*ρ* = − 0.01; *p* > 0.05). Similarly, there was no significant correlation between CN CSA and HS (*ρ* = 0.21; *p* > 0.05) (Fig. 6B), as determined by Spearman's rank correlation coefficient.

## Discussion

The primary objectives of this study were to analyze the ECAPs of ART and AutoART and to investigate the correlations between the respective thresholds and slopes. Subsequently, the ECAP thresholds of ART and AutoART and the ECAP slopes of ART and AutoART were correlated with key parameters such as CN CSA, SI, and HS. Finally, the study was extended to examine correlations between CN CSA and SI, and CN CSA and HS. Although AutoART measurements are widely used in practice due to their many advantages, there is a lack of published studies comparing the AutoART measurement paradigm with ART in adults. Only one of our previous studies compared the two measurement paradigms in infants and children up to 18 months of age [8].

Like our previous study in children, the overall success rate of ECAP responses with AutoART was lower compared to ART in adults. However, the remaining ECAP responses obtained with AutoART showed a biphasic morphology and were of comparable quality to those obtained with ART. The discrepancy in ECAP measurement results between AutoART and ART may be due to the timing of intraoperative measurement. AutoART was performed during wound closure, making it more susceptible to external interference by the surgeon. This may also explain why the ECAP responses of ART and AutoART on the same electrodes were not always congruent in individual subjects.

The Pearson correlation coefficient showed a strong positive significant correlation between the ECAP thresholds for AutoART and ART, with a trend of increasing correlation. Interestingly, the averaged ECAP thresholds for AutoART were lower for the more apical electrodes (E1–E5) compared to the more basal electrodes (E6–E12). This finding is consistent with Brill et al. [31], who used the FineGrain strategy, a precursor to the AutoART paradigm, and also observed lower ECAP thresholds at apical electrodes and higher thresholds at basal electrodes. One possible explanation for this phenomenon is that the distance between the SGCs and the apical electrodes is shorter apically due to the narrower cochlea [21], resulting in a lower threshold for eliciting ECAP [32]. However, this hypothesis cannot be definitely supported for ART, because is no clear pattern of electrode distribution with respect to ECAP thresholds. In most of our subjects, ECAP thresholds were higher with AutoART compared to ART. Studies by Estienne et al. [7] also showed higher ECAP thresholds for FineGrain, the precursor for AutoART, than for ART. One possible explanation for this disparity is that ART uses a linear fit along the steepest portion of the AGF, whereas FineGrain and therefore AutoART uses a sigmoid fit to the AGF, extrapolating linearly from the inflection point of the sigmoid to baseline [6].

In our previous study in children [8], we also found a positive significant correlation between ECAP thresholds, but no clear pattern of electrode distribution for averaged ECAP thresholds.

The Pearson correlation coefficient also demonstrated very strong correlations between the ECAP slopes for ART and AutoART, with a trend of increasing correlation. These correlations were so strong that the test statistics showed no significant differences between the two measurement paradigms, in contrast to the ECAP thresholds. This is promising because the ECAP slope is directly related to the number of SGCs [33, 34], which should not differ when ART and AutoART are measured in the same individual. Strikingly, the averaged ECAP slope of the apical electrodes showed higher values than those of the medial and basal electrodes. This finding is consistent with previous observations from other studies [31, 34, 35].

To compare ECAP responses with SI, we chose audiometric data collected 6 months after initial fitting. Due to the initially high percentage of SI for numbers in our subjects, which in some cases already reached 100%, and the resulting ceiling effect, we considered the SI to be less suitable for comparison with the ECAP responses. The situation was different for monosyllabic SI. Subjects started at a relatively low level and showed improvement over time. For the correlation of the absolute values in SI, we chose the last observation time of 6 months because by that time the subjects had become accustomed to the speech quality of the CI, minimizing the confounding factors that can influence SI [36]. However, we found no significant correlation between the ECAP thresholds and slopes for ART and AutoART and the absolute 6-month SI values. In addition, there was no significant correlation between the ECAP values of both measurement paradigms and HS, which was defined as the difference between baseline and 6-month SI. In other words, SI was independent of whether the patient had a lower or higher ECAP threshold and slope. Cosetti et al. [14] also found no significant correlation between intraoperative AutoNRT ECAP thresholds of 4 selected electrodes and the monosyllabic consonant-nucleus-consonant (CNC) scores in English in their CI-eligible children and adults 1 year post-implantation. Conversely, Pierre et al. [11] found mixed results depending on the CI type between the monosyllabic SI in Swedish (Swedish Clinical Speech Audiometry Test), also 1 year post-implantation, and the intraoperative AutoNRT ECAP thresholds over eight electrodes. Their study showed a significant negative correlation for subjects with a CI512 from Cochlear, but no significant correlation for subjects with a CI532 from the same manufacturer. We suspect that this implant specificity is due to chance. Brown et al. [3] also found no significant correlation between ECAP thresholds measured with NRT precursor nerve telemetry and the Iowa Northwestern University Auditory Test Number Six (NU-6) in English in subjects of unknown age at unknown time points on 8 electrodes. However, they obtained positive significant correlations between the language test and the ECAP slopes. Similarly, Kim et al. [10] obtained relatively strong correlations between NRT ECAP slopes and the CNC Monosyllabic Word Test in adults over up to 8 selected electrodes at unknown time points. Overall, it is currently not possible to predict intraoperatively how SI will develop after implantation based on ART and AutoART ECAP measurements. It should also be noted that the overall range of SI and HS values is relatively wide as it depends on several factors such as duration of deafness, concentration and memory abilities, intelligence, motivation and/or hearing training of the subjects.

In a previous study in children, we performed an analysis of CN and FN CSA using an elliptical formula [8]. Our results showed that, on average, the CN and FN CSA of the children was significantly smaller than that of the adults. These results suggest that the cranial nerves are incompletely developed at birth and subsequently increase in size throughout life. However, we did not find any comparable studies in the medical literature. A notable similar study in adults that also measured CN and FN CSA, was published by Naguib et al. [37]. They found smaller CN and FN CSA than in our study. However, they determined CSA using a circumferential measurement, while we used an elliptical formula with two diameters. We suspect that the discrepancies are due to differences in scanner types, slice thicknesses, and even within our study, there were large variations in CN CSA among individual patients. Another factor is the method of measurement itself. A circumferential measurement provides more accurate results than an elliptical formula measurement when the course of the nerve is irregular, as the CN and FN are not uniformly round or elliptical in every patient. In addition, a 3D CISS and bFFE sequence cannot differentiate between the nerve and its surrounding myelin sheath, leading to different delineation of the nerve by each observer.

Preoperative CN CSA determinations could also be used to predict the outcome of intraoperative ECAP measurements. Except for our previous study in children [8], there are no comparable studies investigating this. However, in both children and adults, there was no significant correlation between ECAP measurements for ART and AutoART and CN CSA. This suggests that current MRI images are not predictive of whether a thicker/thinner CN will result in a lower/higher ECAP threshold or a flatter/steeper ECAP slope. Since the CN CSA is directly correlated with the number of SGCs [21], it would also be interesting to investigate whether there is a relationship between CN CSA and the SGC population in these individuals. However, such studies can only be performed postmortem. Although Nadol et al. found significant correlations between the number of SGCs and CN diameter, the large variability in diameter in his study makes predictive evaluations difficult [21].

Furthermore, our study did not confirm the hypothesis that CN CSA correlates with monosyllabic SI. This was true both for the absolute postoperative SI at the 6-month follow-up and for the HS. This means that patients with a smaller CN CSA may still achieve a good SI or patients with a larger CN CSA may experience a worse SI. To date, there is no study that relates monosyllabic SI to CN CSA. Some studies have evaluated SI by postmortem histological examination of SGCs. However, most of these studies had relatively small sample sizes, limiting the significance of their findings [24, 38–40]. Only Khan et al. [23] had a relatively representative number of subjects (*n* = 14). Nevertheless, they found no significant correlations between word recognition on the NU-6 and the number of SGCs. It would be interesting to figure out if this observation holds true for the SGC of our subjects.

## Conclusions

In conclusion, our study shows that AutoART provides similar ECAP measurement results to ART in adults. However, our study was limited by the fact that the influence of CN on intraoperative ECAP measurement and speech perception could not be investigated, and current MRI scanners are limited. In addition, objective electrophysiological ECAP measures cannot accurately predict hearing status and speech perception in implanted patients. Therefore, further research is needed to better understand and develop both the influence of ECAPs on speech perception and the influence of CN on ECAP measurements and speech perception.

## Data Availability

Anonymized version of the data is available upon request.
